# Supplementary material for: Molecular Profiling of Lymphatic Endothelial Cell Activation In Vitro
Source: Int J Mol Sci. 2023 Nov 22;24(23):16587. doi: 10.3390/ijms242316587 (PMC10706153; doi:10.3390/ijms242316587)
Supplement: Supplementary file 1 [file ijms-24-16587-s001.zip › ijms-2710685-supplementary.pdf]

Supplementary Table S1. The most downregulated genes in common between FVS and co-culture treatment are reported.

GENE symbol	log2FoldChange co-culture vs Ctrl	padj co-culture vs Ctrl	log2FoldChange FVS vs Ctrl	padj FVS vs Ctrl
LOC102723996	-2.652057279	0.047185414	-8.107897007	1.77039E-06
GRIN2B	-4.436279171	0.012079183	-5.523810991	0.003431689
IGSF23	-1.653032409	0.011730151	-3.921890457	3.54621E-08
CX3CL1	-1.60055391	2.5341E-11	-3.903330707	1.53826E-50
ZSWIM2	-2.931620957	0.001418986	-2.416260257	0.009041778
TMEM37	-1.727477495	0.002321872	-3.455965541	7.65742E-09
LTB	-1.313228061	1.02059E-06	-3.67318213	3.54881E-36
TNFSF18	-2.852094676	2.1497E-139	-1.830594257	5.46911E-61
CRYAB	-1.761501324	8.01336E-08	-2.824051014	1.27133E-17
GDF3	-2.25735164	1.53996E-06	-1.833976611	0.000117063
TRIM59-IFT80	-2.257509274	0.003656921	-1.796970507	0.025477852
TACSTD2	-1.203036521	9.01141E-42	-2.807576226	4.8316E-186
ANKRD1	-0.406152618	3.14226E-05	-3.49898096	0
GJA4	-2.016322106	4.60512E-75	-1.858236425	5.84834E-64
GPC4	-1.460854699	9.33127E-09	-2.381581261	1.15012E-19
ANKRD65	-1.411557058	0.026152411	-2.011619203	0.001472629
TCIM	-0.436361897	0.001087874	-2.967243838	1.6847E-127
TNFRSF9	-1.416801592	9.23268E-06	-1.934862145	6.83447E-10
HTR2B	-1.482927687	3.47755E-07	-1.817127987	4.24869E-10
GDF7	-0.950259865	1.36324E-12	-2.273561592	2.25042E-61
COLEC10	-2.074466278	1.1583E-05	-1.143057075	0.023178763
ICAM1	-0.263428222	0.005523988	-2.882724005	5.8395E-234
SERPINE1	-0.955729496	4.85491E-54	-2.075382064	7.5687E-253
SLC2A12	-0.38654115	6.49165E-08	-2.541637629	1.0906E-293
TNFRSF11B	-1.008603107	1.09595E-06	-1.895020545	3.68922E-20
GBP4	-0.623957173	0.006829667	-2.240521346	3.18607E-25
EDN1	-0.168600384	0.010216946	-2.536180001	0
CCDC85A	-1.206839866	1.01853E-06	-1.465987844	3.24533E-09

HBEGF	-1.158008402	6.46438E-43	-1.502951313	1.10713E-69
NPIPB2	-1.09240646	0.02933852	-1.562219298	0.000950467
TNFSF15	-1.901123328	2.30992E-36	-0.751070246	2.43007E-06
RFPL4AL1	-0.993607867	7.56803E-07	-1.576955524	2.37518E-15
INSYN2B	-1.037012714	1.02321E-25	-1.518550213	1.64646E-53
CCN2	-0.422191357	1.23668E-15	-2.119802055	0
FADS3	-0.751068322	2.47864E-21	-1.783886561	2.1951E-112
IFIH1	-0.386052162	0.009048156	-2.124014838	1.82026E-58
DIRAS3	-1.169084195	0.029729562	-1.337522919	0.011953266
SLC7A14	-0.876224656	2.97697E-09	-1.619687648	7.87834E-26
EID3	-0.795026547	0.0037454	-1.606603316	9.51381E-10
ADRB1	-0.392332564	2.90497E-05	-2.008544153	3.4185E-104
KCTD8	-0.762368336	0.021100078	-1.570600002	2.97598E-07
NAPRT	-0.749223519	0.041929466	-1.56430713	2.18743E-06
DHRS3	-0.528735622	1.06242E-06	-1.595057394	3.34178E-54
ACKR3	-0.900027427	9.43958E-22	-1.164961935	7.94244E-36
SDC4	-0.24642683	0.007349457	-1.812277116	2.9619E-110
PNMA6A	-1.000003679	0.035742054	-1.050016481	0.029802388
SOX17	-1.086409447	2.05169E-30	-0.938800248	1.20781E-22
PRRG4	-0.647640153	3.94907E-05	-1.335928398	3.08351E-18
DDIT4L	-0.923520053	2.16713E-13	-1.015481527	6.33746E-16
DRAXIN	-0.918664547	1.89584E-18	-0.996394598	2.78108E-21
CITED4	-0.699059279	0.014387041	-1.200459233	1.11838E-05
LCAT	-0.825245872	0.004208632	-1.066987525	0.00016312
NUDT4B	-0.367389308	6.25849E-05	-1.495382295	2.23148E-68
ZSWIM5	-0.674881968	2.84795E-19	-1.174199687	7.16669E-56
BOK	-0.504338423	1.36131E-12	-1.320256881	4.37504E-80
SQSTM1	-0.538749252	1.66262E-12	-1.239697744	1.38162E-63
PLA1A	-0.332971025	0.003126213	-1.404381029	4.79376E-44
AGMAT	-0.944382185	2.84227E-05	-0.732280866	0.001886592
BMP6	-0.370842219	1.5466E-11	-1.29670634	1.8658E-131
RFPL4A	-0.501456942	0.003717092	-1.145676725	6.17118E-13

APBA1	-0.812646721	2.54668E-08	-0.802776322	4.87617E-08
FBXO32	-0.73070521	0.000451545	-0.88419372	1.22045E-05
PRSS12	-0.609621293	4.96899E-06	-0.941579099	2.27157E-13
MRAP2	-0.828977291	3.56138E-07	-0.696091256	2.91991E-05
TXNDC5	-0.189504117	0.000376493	-1.31090758	4.7943E-169
TRMT9B	-0.267313434	0.048260934	-1.214286039	9.53047E-27
JAG2	-0.878537365	1.13764E-15	-0.58314019	2.95411E-07
IRF2BPL	-0.315431371	0.042243957	-1.098962404	3.73989E-16
STARD10	-0.482240502	0.001989178	-0.887050405	1.24249E-09
SYDE2	-0.750293477	0.000592702	-0.618190109	0.006407632
PLXNA4	-0.800554581	9.24905E-16	-0.559183069	4.11622E-08
DNAJB4	-0.623265506	5.60051E-28	-0.722179406	1.72222E-37
ZXDB	-0.810347048	4.8239E-16	-0.527825561	2.96674E-07
TMEM140	-0.510949403	1.00336E-12	-0.814398415	4.1465E-31
ZNF702P	-0.523304345	5.11031E-08	-0.790279027	2.76821E-17
DTX4	-0.286609866	0.000723272	-1.025752766	2.43992E-41
CCND1	-0.552550635	1.5673E-36	-0.744323242	4.57506E-66
SEMA3G	-0.802428335	6.82643E-24	-0.477669452	6.42768E-09
SLC5A3	-0.70121905	1.87879E-09	-0.578753041	1.1485E-06
ABR	-0.576486454	0.000447732	-0.701933273	1.0494E-05
SOX7	-0.66785283	1.01853E-06	-0.607301612	1.2689E-05
ZNF350	-0.634260162	0.001900498	-0.623274968	0.002471477
IGFL4	-0.602489535	8.82216E-06	-0.6161862	5.50885E-06
C1orf115	-0.240482993	0.000397975	-0.967171986	1.0791E-54
OR10H1	-0.663827098	0.001535219	-0.503978685	0.023780622
HMOX2	-0.818000931	1.95909E-13	-0.349523135	0.004811288
SIK2	-0.641742973	8.4949E-18	-0.518446326	7.36003E-12
TMSB15B	-0.683168401	2.87002E-06	-0.475556793	0.00207945
TMEM150C	-0.542001563	0.007956161	-0.61376909	0.002215967
LATS2	-0.475314708	4.38626E-12	-0.675966008	1.27778E-23
GIPC2	-0.306902642	0.003778181	-0.841113958	2.33113E-18
FOXC2	-0.492672846	0.013968705	-0.655047366	0.00069864

ABCC1	-0.52789997	7.60476E-05	-0.608187102	3.65992E-06
CCL5	-0.75484721	1.00203E-07	-0.372538164	0.018989307
TAS2R20	-0.552629546	0.013097163	-0.55931401	0.012905337
CCND2	-0.921223031	5.08867E-59	-0.189570178	0.003715229
PLCL2	-0.5915797	4.46768E-07	-0.515304809	1.61036E-05
SYT11	-0.177517406	0.028983771	-0.928827864	4.44928E-41
CHST2	-0.692845539	1.71376E-05	-0.389195164	0.0311779
B3GLCT	-0.589535343	4.73607E-22	-0.49182908	1.47103E-15
ARHGEF28	-0.696255076	7.16409E-30	-0.373837436	4.22363E-09
CAVIN2	-0.686223418	1.26873E-41	-0.379485218	3.5854E-13
ZHX2	-0.583463983	9.46121E-05	-0.472536412	0.002464814
GOLGA6L17P	-0.466836759	0.025420087	-0.586167891	0.003648688
TSPYL5	-0.421366675	2.86881E-05	-0.629359568	1.17811E-10
COG5	-0.591120932	3.91573E-09	-0.45747924	8.99821E-06
ARFGEF3	-0.4413246	0.003340429	-0.601672349	3.07344E-05
FAM200B	-0.321951722	0.000596839	-0.710643753	2.6456E-16
NUPR1	-0.335322756	0.007372469	-0.689454266	1.28064E-09
CYP27A1	-0.286312537	0.0188251	-0.723353419	1.7247E-11
LRRC37A2	-0.466959544	0.044791185	-0.530216481	0.019707827
OR10K1	-0.425569472	0.013108928	-0.562664308	0.000635942
BTG2	-0.232922991	0.020036773	-0.750632405	6.39502E-18
KCTD12	-0.43053993	2.42586E-22	-0.551020582	1.88472E-36
RPS9	-0.470996655	1.97774E-10	-0.48864662	3.58383E-11
ANKRD36BP2	-0.46978417	2.4717E-05	-0.489030838	1.03627E-05
THBD	-0.574727771	4.40105E-07	-0.381541619	0.001741361
SLC2A6	-0.627758965	2.44846E-09	-0.3264555	0.004694347
RHOD	-0.416143179	0.023110877	-0.534365402	0.002567035
LY96	-0.406148774	0.000574503	-0.540320226	2.17887E-06
SMG1P1	-0.566390002	4.89558E-06	-0.379192708	0.004232457
PHETA2	-0.500193274	0.000587953	-0.442076567	0.003185157
FZD1	-0.377253323	0.030085214	-0.549386225	0.000815458
B3GNT9	-0.420372494	0.02396624	-0.504322215	0.005659647

FAM110D	-0.479553225	0.02042599	-0.44170396	0.040158825
C4orf36	-0.475094903	0.006014079	-0.442914466	0.012206145
C1QTNF5	-0.55495155	4.52199E-14	-0.359425834	2.85055E-06
DUSP16	-0.585269442	4.41783E-05	-0.321084702	0.048630305
TMTC2	-0.260757831	0.010244395	-0.628650022	4.28596E-12
TBC1D22B	-0.437752717	2.19145E-05	-0.445176964	1.6534E-05
TBC1D9	-0.594333117	2.34489E-22	-0.279136138	1.80284E-05
LOC389831	-0.485574112	9.53402E-10	-0.376206368	3.96028E-06
HS6ST1	-0.390589518	0.001441371	-0.459942752	0.000147084
SHISA9	-0.525375378	1.19055E-08	-0.32181906	0.001114733
ZNF697	-0.556630248	4.44771E-12	-0.290427139	0.000884053
OR4F17	-0.347876192	0.022232872	-0.475716891	0.000989889
TM4SF18	-0.620019559	4.29009E-30	-0.187865622	0.002198206
DUSP3	-0.473102869	6.24578E-20	-0.333765009	2.97867E-10
SLC26A2	-0.197210109	0.003377513	-0.597098681	2.62453E-23
GDF15	-0.501993495	2.52805E-10	-0.287345737	0.000806612
PCDHGA6	-0.443055594	0.001832442	-0.33588461	0.027328864
TRIM47	-0.408915215	0.001068678	-0.368091155	0.004017301
UVRAG	-0.503893413	6.55654E-15	-0.265161758	0.000125392
TMEM212	-0.384337231	0.001834258	-0.384141206	0.001967671
B4GAT1	-0.278511623	0.014877937	-0.487998867	3.73494E-06
RAB33B	-0.473108921	2.59374E-08	-0.281227893	0.00216234
PCBD1	-0.305423235	0.041415445	-0.445592709	0.001329074
CYSLTR1	-0.361718706	0.008939143	-0.380438938	0.005692599
VKORC1L1	-0.423770132	2.70701E-07	-0.263065809	0.003087813
FRK	-0.44000734	1.37206E-07	-0.245402962	0.007309215
HOXD10	-0.42605382	6.30568E-05	-0.25653484	0.030253271
OR5AS1	-0.385623979	0.005368821	-0.295773606	0.046758024
OR14J1	-0.408805775	6.04885E-05	-0.254088298	0.023574907
ACTN4	-0.318131003	2.75587E-05	-0.32075629	2.40521E-05
MED13L	-0.319737254	0.003202063	-0.297206803	0.007032231
DDAH2	-0.375353152	0.000275589	-0.233068499	0.043152298

TSPAN12	-0.376718796	1.83682E-07	-0.230245337	0.003160437
SYNRG	-0.30824816	0.015466084	-0.29577764	0.022344538
JUN	-0.236053599	0.002597459	-0.340085051	4.75358E-06
BCL7A	-0.287979691	0.030880534	-0.287336907	0.033485076
TRAM2	-0.316224757	0.000891379	-0.25545949	0.010327062
SULT1B1	-0.215206659	0.01500565	-0.351529696	1.54717E-05
PDXDC1	-0.299297586	0.003463169	-0.240005332	0.026399219
ATF6	-0.283646728	9.61784E-08	-0.241597448	8.19429E-06
WASH5P	-0.272362958	0.00425696	-0.244858654	0.012795081
POLR2M	-0.182684106	0.043641027	-0.322698589	6.56117E-05
GTF2IP4	-0.275485021	9.01244E-07	-0.229889715	6.39694E-05
LAPTM4A	-0.202777843	0.039124554	-0.295345685	0.00114402
MLLT6	-0.296116236	0.001381808	-0.20118448	0.049836405
MANSC1	-0.273128483	0.006968659	-0.222718437	0.037743554
CD2AP	-0.267510172	2.06697E-05	-0.224855537	0.0005248
CRCP	-0.234682737	0.030362184	-0.251647339	0.019543691
STK38L	-0.275121497	4.71454E-05	-0.186050026	0.010759274
SSR3	-0.206024397	0.002313549	-0.196743725	0.003961479
CTTN	-0.247667012	3.72798E-06	-0.123883101	0.044271827

Supplementary Table S2. The most upregulated genes in common between FVS and co-culture treatment are reported.

GENE symbol	log2FoldChange co-culture vs Ctrl	padj co-culture vs Ctrl	log2FoldChange FVS vs Ctrl	padj FVS vs Ctrl
GJA3	8.196381564	8.66787E-07	4.188689603	0.034993106
FAM72C	5.753090777	9.60302E-16	2.529597081	0.001852244
GPR3	4.721040899	0.000437508	3.341519049	0.023486515
IL13RA2	5.634887333	2.0315E-166	1.263390248	9.19912E-08
KIF11	4.030691135	5.5971E-137	1.778467361	1.0766E-25
IGFBP6	4.385796301	3.55972E-42	1.288516804	0.001038095
TMEM158	2.811597223	4.47923E-05	2.632150848	0.000187124
H3C8	4.515219932	5.36033E-73	0.73904011	0.02280819
H3C15	3.874701053	5.6411E-197	1.279949842	1.21524E-20
H2BC9	4.27989137	1.06472E-83	0.600972647	0.043357723
H3C12	3.81324413	1.70028E-80	0.81040955	0.000738911
H2AC12	3.425806621	8.22654E-94	1.143056623	3.2927E-10
BRIP1	3.305053579	2.42735E-11	1.258138221	0.036199474
H3C7	3.569515866	5.03237E-46	0.969042975	0.000854765
C1R	3.815743178	7.98471E-53	0.719331918	0.024193788
DEPP1	2.002615044	2.0074E-223	2.473536576	0
SOCS3	2.284862278	2.5059E-133	2.062089674	1.5322E-107
H2BC17	2.975195636	1.26251E-66	1.325426161	3.66203E-13
H2BC3	3.55418925	5.48424E-52	0.72683819	0.010789241
ERCC6L	3.203672297	7.10774E-35	0.987111873	0.001194632
H3C3	3.001671786	9.52482E-90	1.132579209	1.01419E-12
H2AC14	3.448595537	1.20187E-74	0.685628848	0.002709374
H3C2	2.79268409	5.84817E-90	1.300992639	1.80498E-19
H3C4	2.887519268	2.59575E-89	0.927977874	4.71754E-09
H2BC13	3.263117512	1.57865E-81	0.496560422	0.023533023
RGS16	2.02595411	3.74588E-07	1.719880011	2.90747E-05

H2AC13	2.903062783	2.6782E-112	0.826236177	5.22931E-09
KCNJ2	2.664101422	7.67326E-13	1.056493886	0.018003475
H2AC11	2.772825099	4.83922E-68	0.902500544	1.84483E-07
LENG8	1.672417402	0.000974506	1.971995434	6.67706E-05
SDHAF3	2.087325832	2.46282E-13	1.425962242	2.43007E-06
CCNB2	2.878454668	8.80031E-67	0.589833742	0.003609124
H2BC11	2.332882163	2.5928E-49	1.020180925	1.03543E-09
KIFC1	2.318663987	3.89793E-14	0.994933108	0.004152556
H2AC18	2.619389666	3.1536E-123	0.693612782	6.16314E-09
CCN3	2.274260271	1.53576E-14	1.006992202	0.003038123
CCL14	0.58020211	3.57144E-06	2.671697271	1.579E-130
H1-5	2.002212184	3.40638E-60	1.245316809	3.17757E-23
H2BC7	2.782488219	6.45496E-57	0.463037332	0.040763297
GPC1	0.570953524	6.0815E-05	2.650720447	2.2134E-101
TRIB1	0.64568158	4.14783E-05	2.485986817	1.34597E-70
KIF18A	2.463568453	1.17703E-27	0.623783212	0.028574935
DUSP4	0.937979953	1.06913E-25	2.015130534	8.6043E-120
H2AC21	2.443234801	8.67721E-60	0.48538467	0.008514493
NCR3LG1	1.465720614	8.14771E-13	1.421256469	4.3235E-12
HS3ST3B1	1.177291212	0.000591438	1.704548788	1.19953E-07
ASF1B	2.302199319	1.57969E-29	0.521846212	0.04508194
ITGB3	1.086641064	6.40471E-70	1.722283568	1.4373E-177
H2AC4	1.951428919	1.11243E-27	0.738348905	0.000251581
H2AC15	1.809655472	1.52737E-16	0.879957166	0.000312701
FEN1	2.156640059	3.10476E-63	0.471017266	0.001804718
CIC	0.975294945	0.000169187	1.631729823	1.91041E-11
H2AC16	1.487157372	9.05869E-12	1.070158223	3.15643E-06
BHLHE40	0.586593606	1.31046E-07	1.960017806	4.20531E-82
DUSP5	0.742983235	8.22679E-25	1.790565445	4.9645E-148
SHCBP1	1.943820539	1.82501E-23	0.548266168	0.019176893

JUNB	1.002285549	1.6837E-14	1.436469863	2.53341E-29
FASN	0.612374698	0.000422555	1.818834851	2.30559E-31
LDHA	1.225032256	1.878E-54	1.163017696	2.7719E-49
DUSP6	0.691038363	1.57139E-31	1.669310337	1.4124E-184
MELK	1.820624593	8.08797E-37	0.532060284	0.001400393
H2AC17	1.954257023	2.30201E-37	0.388942037	0.043739189
RGS17	0.64058082	2.36322E-05	1.698938925	2.37441E-35
DBF4	1.49418846	3.62475E-41	0.817808339	2.6461E-12
HASPIN	1.470138954	7.69371E-11	0.775943271	0.002106513
TC2N	0.87173313	9.87685E-11	1.227787162	6.57954E-21
NAMPTP1	1.182900475	6.97041E-16	0.902535228	2.08184E-09
ANKRD37	0.912252428	8.43128E-09	1.113878039	7.08457E-13
H2BC15	1.219092194	1.03389E-18	0.804851602	2.14553E-08
HLA-DPA1	1.26318336	4.73457E-17	0.729033874	5.40941E-06
METRN	1.053290903	9.19866E-05	0.921019359	0.000884254
PPAT	1.264072692	2.46395E-36	0.578727932	7.72212E-08
H2BC12	1.253709782	3.091E-27	0.585818942	2.18312E-06
MCM6	1.171609141	3.50372E-25	0.532970826	1.39449E-05
ARRDC3	0.517567041	1.74254E-08	1.186710469	1.47653E-42
H2BC5	1.200940874	4.28063E-19	0.492323758	0.001028302
UBALD2	0.791816827	3.41944E-05	0.894542594	1.94504E-06
TGFB3	0.806010922	7.67815E-05	0.84612953	2.93432E-05
RHPN2	1.037948977	2.34522E-11	0.561769857	0.001032438
EIF4EBP1	0.734790236	9.62871E-12	0.858786015	8.56414E-16
POMP	0.569069591	2.84809E-14	0.955488988	1.14873E-39
DDIT4	0.960244905	1.96364E-29	0.551043842	7.35105E-10
MYCBP	1.050334877	3.24459E-10	0.459401426	0.017032222
ARG2	0.49299536	2.90497E-05	0.961844745	1.89108E-18
C1QBP	0.88002574	9.08765E-22	0.567179369	2.18866E-09
MLLT11	0.939264694	1.52439E-12	0.457674486	0.002096176

PAICS	0.999937064	3.89316E-36	0.39673688	3.51493E-06
TCF19	0.947358942	2.0154E-07	0.439106517	0.040158825
COTL1	0.288186026	7.1852E-06	1.095122932	6.26905E-78
BOP1	0.753866738	7.07056E-13	0.569075726	1.74591E-07
RHOU	0.938351105	9.66604E-39	0.381541229	9.38312E-07
RRP15	0.923026427	6.13894E-12	0.37063935	0.016853456
LRRC58	0.709909583	8.932E-25	0.58190038	9.27074E-17
MEOX2	0.915778754	3.35754E-13	0.359581036	0.014772452
CSPG4BP	0.672170927	1.0155E-10	0.58443289	3.36486E-08
PRKAR2B	0.43830304	0.000793876	0.810465342	1.4848E-11
NME1	0.509247259	3.90276E-08	0.720113973	1.05075E-15
MRTO4	0.789812355	1.14664E-18	0.432821635	5.65814E-06
TUBB4B	0.868136615	8.25136E-33	0.328999157	3.18065E-05
IPO4	0.450753201	2.51217E-05	0.734375698	4.01888E-13
SNRPA	0.607544436	1.94543E-10	0.575270164	2.43471E-09
MIF	0.544075753	1.20138E-07	0.637442254	3.24101E-10
EXOSC4	0.763155807	4.59868E-07	0.414877739	0.016176023
SSRP1	0.75616192	1.1E-40	0.419339264	9.43366E-13
NHP2	0.583845018	2.56223E-10	0.583532052	2.91634E-10
MRPL3	0.817487878	1.53917E-25	0.345801236	5.1288E-05
C11orf98	0.861683778	5.79087E-12	0.301182954	0.047632272
RUVBL1	0.861217055	3.82958E-21	0.286232683	0.006688634
BOLA2B	0.743595321	3.17323E-09	0.39583209	0.004641236
FBL	0.802542125	1.44224E-21	0.330468697	0.000426668
NUDT15	0.708727259	3.81262E-08	0.408703566	0.00381316
TOMM34	0.814595351	7.66354E-25	0.302558417	0.0006907
TFB2M	0.777588761	1.36423E-12	0.3386091	0.007032231
MRPS12	0.536924994	0.000263583	0.577011638	8.27623E-05
YRDC	0.831677903	2.01805E-14	0.274430617	0.038527071
SOCS7	0.609985872	0.000343024	0.495033141	0.005354927

AATF	0.726513277	5.51372E-19	0.368177903	2.88258E-05
RRS1	0.670172466	5.4214E-05	0.409926688	0.028574935
TIMM8A	0.607254261	0.00051266	0.463953886	0.013275845
SLC25A5	0.526615194	7.97405E-14	0.536717216	2.3958E-14
DNAJB1	0.666333412	8.30924E-09	0.372708364	0.003242697
RBM28	0.437657193	2.56444E-05	0.597865726	2.36767E-09
PGAM5	0.685997169	2.64564E-18	0.295751302	0.000734285
HNRNPR	0.612067954	1.73317E-16	0.369155498	2.14656E-06
TEAD4	0.642935439	1.28393E-07	0.318376855	0.022713326
NDUFAF4	0.481472062	8.65962E-05	0.476218981	9.16194E-05
PIM3	0.358705049	1.09798E-07	0.584192413	2.38972E-19
PAK1IP1	0.620517354	1.86616E-07	0.319064374	0.018310629
TUBG1	0.531413948	2.4092E-08	0.404925697	4.32719E-05
RPS5	0.607536268	3.03374E-10	0.314754356	0.003001951
H2BC4	0.581722231	2.70757E-06	0.339997552	0.013103705
PRDX6	0.547674496	3.0161E-15	0.357467725	7.53078E-07
UBE2M	0.520684599	3.5074E-09	0.383832914	3.05003E-05
BOLA2-SMG1P6	0.462237748	4.51894E-05	0.441432244	0.000120917
SOCS5	0.573428322	9.05443E-15	0.32530085	3.66242E-05
CYCS	0.48724118	1.83814E-15	0.404753691	7.51292E-11
ATP5F1B	0.405469533	2.74013E-12	0.480446202	4.62599E-17
TOP1	0.499196662	1.15864E-05	0.366760344	0.002367059
EIF5A	0.295049578	0.000345205	0.567927708	1.03315E-13
NDUFB9	0.436714581	2.97696E-05	0.410287098	0.000109849
RBM8A	0.51669644	3.08273E-08	0.321894765	0.001303428
EIF2S2	0.428550891	6.49347E-11	0.402821181	9.94662E-10
CHORDC1	0.40852132	2.10057E-09	0.421091525	6.27995E-10
COX8A	0.517847765	2.7427E-07	0.306014272	0.005533883
EIF6	0.57537225	9.4113E-13	0.246813001	0.006688634
MRPL13	0.495767795	7.86348E-06	0.303207097	0.013463882

TRMT10C	0.404649734	2.98607E-05	0.383841121	9.24425E-05
OXSR1	0.326666146	6.68295E-06	0.461279027	3.7917E-11
TOMM70	0.537683993	1.02459E-16	0.250248948	0.000426668
RPS2P5	0.405262532	7.03728E-05	0.378166278	0.000263398
GADD45GIP1	0.410428869	0.001025402	0.366326499	0.004438602
SMIM15	0.432040078	1.55608E-05	0.332644385	0.001531155
ALYREF	0.418472572	0.001068678	0.326144783	0.016176023
PRPF19	0.357821806	3.20261E-07	0.379460752	5.18257E-08
XRN2	0.340881367	3.20261E-07	0.371817216	1.83383E-08
GLUD1	0.316397742	0.000933405	0.392475065	2.10412E-05
TSEN34	0.383067065	0.000735827	0.32258727	0.006438515
RLF	0.264139072	0.001133913	0.441106216	4.93016E-09
RARS1	0.376069885	1.23824E-08	0.328240453	9.9814E-07
RIOK1	0.414269401	9.78463E-05	0.284616213	0.014082454
PPP1R15B	0.445546798	9.77375E-13	0.240767614	0.000386244
SYPL1	0.423364207	3.35416E-11	0.247599132	0.000305042
ALDH1B1	0.400450583	6.46154E-05	0.260459238	0.018639545
GLO1	0.309459305	0.00019277	0.341865818	2.91991E-05
RPS25	0.3778809	3.23088E-05	0.27012802	0.005220235
TBCE	0.380014048	0.000197086	0.240146251	0.035248143
SLC35G2	0.366933222	0.00077896	0.251755228	0.036199474
MRPL50	0.304948755	0.001246719	0.282621609	0.003370646
SRSF9	0.267506245	0.001333427	0.312439209	0.000122211
PRDX3	0.34567555	8.6391E-05	0.214091507	0.028287999
CST3	0.221260098	0.000682088	0.321512285	2.07214E-07
PPIB	0.288316877	7.34821E-05	0.247742631	0.00095518
RPL7A	0.239124456	0.001014184	0.273498691	0.000120538
RPL39	0.258559894	0.000317829	0.204920173	0.006408501
PRKDC	0.204790263	0.000757123	0.207212415	0.000665328
